# The neurobiology of oral cancer pain

**DOI:** 10.1186/1744-8069-10-S1-O14

**Published:** 2014-12-15

**Authors:** Brian L Schmidt

**Affiliations:** 1 Department of Oral & Maxillofacial Surgery, NYU College of Dentistry, New York, NY 10010, USA

## 

Oral cancer pain is more severe, on average, than pain from any other cancer. The public health problem of cancer pain is, ironically, exacerbated by improved chemo- and radio-therapies that prolong survival. The intensity of oral cancer pain escalates with disease progression; terminal patients generally experience debilitating pain during their final months of life. The etiology of oral cancer pain is not known and current treatment is ineffective. Cancer pain is hypothesized to result from a tumor-mass effect and/or activation of primary afferent nociceptors by mediators liberated by the cancer. Dr. Schmidt discussed the molecular cross-talk between cancer and peripheral nervous system that might responsible for pain. He presented data demonstrating a reciprocal proliferative effect between cancer and surrounding sensory nerves.

